# Urban heat in Johannesburg and Ekurhuleni, South Africa: A meter-scale assessment and vulnerability analysis

**DOI:** 10.1016/j.uclim.2022.101331

**Published:** 2022-12

**Authors:** Niels Souverijns, Koen De Ridder, Nele Veldeman, Filip Lefebre, Frederick Kusambiza-Kiingi, Wetu Memela, Nicholas K.W. Jones

**Affiliations:** a Environmental Modeling Unit, Flemish Institute for Technological Research (VITO), Mol, Belgium; b PlanAct, Johannesburg, South Africa; c Global Facility for Disaster Reduction and Recovery, The World Bank, WA, USA

**Keywords:** Urban heat island, Heat stress, UrbClim, Heat monitoring, Johannesburg, Ekurhuleni

## Abstract

Heat stress is an important threat for human health and urban areas are affected at higher rates compared to rural environments. Additionally, climate change will increase the vulnerability towards urban heat stress in the future. Current high-resolution urban heat stress assessments are limited in time and space due to the high computational costs. In this paper, the UrbClim numerical model is used to simulate urban heat accurately at a fast rate and high spatial resolution for the cities of Johannesburg and Ekurhuleni, South Africa. Using detailed terrain information, (future) urban heat stress assessments are provided at 30 m resolution for both city agglomerations, while meter-scale simulations are executed for a selection of neighborhoods. These model simulations are evaluated using an extensive monitoring campaign in which the local community was heavily engaged. Distinct spatial differences in the urban heat island effect are observed, with greatest heat stress in areas with high building densities and low vegetation numbers. These areas are often characterized by lower socio-economic living conditions. The meter-scale analysis further shows the importance of shade provided by vegetation to lower heat stress in both present and future climate. These assessments offer assistance in the design of climate-resilient urban planning strategies.

## Highlights

• 30 m resolution city-wide heat stress indicators are provided for the past & future.
• Meter-scale analyses of heat stress indicate vulnerable regions.
• Effect of vegetation and building density on heat stress is quantified.
• The implementation of urban green is an effective climate resilient urban planning strategy.
• A monitoring campaign is executed to support modelling results.

## 1 Introduction

Global temperatures have been rising continuously during the last decades. In parallel, heatwaves occur with an increased frequency, intensity and duration and this rise is expected to continue towards the future (Perkins-Kirkpatrick and Lewis, 2020). Urban agglomerations exacerbate heat (the so called urban heat island (UHI) phenomenon) due to for example the concentration of building materials, asphalt surfaces and limited urban green, among others (He et al., 2021b). As such, temperatures can be several degrees warmer inside cities compared to rural surroundings, that are characterized by sparse settlements and more open spaces (Arnfield, 2003). Furthermore, there are many interactions between heatwaves and UHIs, which synergies are not fully disclosed (He et al., 2021a).

Research on the UHI is currently mainly focused on mid-latitude cities from the northern hemisphere, while developing and southern hemisphere areas are often neglected (Roth, 2007, Stewart, 2011, Zhou et al., 2019). Cities in Africa are already prone to heatwaves in present times and will become even more vulnerable towards the future, both due to climate change (Russo et al., 2016, Rohat et al., 2019, Harrington and Otto, 2020, Trisos et al., 2022) and city growth caused by increasing population numbers (Hoornweg and Pope, 2017). In addition, poor housing conditions, such as those typically occurring in dense informal settlements in many African cities, may lead to enhanced heat exposure compared to developed urban areas (Pasquini et al., 2020). Studies over African cities show spatial expansions of the UHI in the last decades (Li et al., 2021) and project a large increase in the number of people that are affected by heat stress towards the future (Rohat et al., 2019, Marcotullio et al., 2021).

Towards the future, it is expected that cities will experience twice as much heat stress than rural areas (Wouters et al., 2017, Marcotullio et al., 2021). The impact of excessive heat exposure on public health has already been observed in the past, with surplus mortality during heatwaves (Kovats and Hajat, 2008, Li et al., 2015) and is expected to increase in areas that already experience heat stress in present times (Gasparrini et al., 2017). Apart from public health, the economic impact of heat stress on labor productivity is projected to be several percentages of the worldwide Gross Domestic Product (GDP) (ILO, 2019, Zhao et al., 2021). Moreover, studies found positive relationships between heat stress and violence over several areas around the world (Chersich et al., 2019).

The UHI has been studied for many years (e.g. Oke, 1988) using different approaches. Thermal land surface temperature measurements from satellites permit sub-kilometer resolution mapping of urban environments and their surroundings (e.g. Zhou et al., 2019, Rodrigues de Almeida et al., 2021). This approach allows to disentangle the drivers of urban heat and the definition of the relation between urban green and temperatures, which has now been established for cities around the globe (Peng et al., 2012). However, there is a large trade-off between spatial and temporal resolution, leading to limited detail in time or space. Numerical modeling of the UHI overcomes these problems. Most studies use meso-scale models, having the advantage of providing a detailed physically-resolved representation of atmospheric dynamics, which can be used both for present-day and future studies. A disadvantage of these models is the high-computational cost, needing several internal nesting steps to downscale driving models and preserve internal variability, limiting their application to kilometer-scale analyses (Kwok and Ng, 2021). Micro-scale and building-scale models on the other hand (e.g. Tominaga et al., 2015) offer very detailed representations of urban canopy and airflow, but are often limited to areas covering a few hundred meters (Mirzaei, 2015).

In order to tackle the problems outlined above, the UrbClim numerical model was developed, which will be applied in this study (De Ridder et al., 2015). UrbClim is a fast urban boundary layer model which permits long-term city-scale urban climate runs at a resolution of hundreds of meters. Due to its fast run-time, it is well suited for simulations of several months to years. Moreover, UrbClim produces similar results as more sophisticated mesoscale models (García-Díez et al., 2016). The model (components) have previously been validated on a variety of cities around the globe, e.g. Ghent, London, Colombo, Delhi, among others, yielding satisfactory results (Lauwaet et al., 2015, Zhou et al., 2016, Hooyberghs et al., 2019, Maheng et al., 2019, Sharma et al., 2019, Caluwaerts et al., 2020).

Research on heat stress has been executed in the past over the cities of Johannesburg and Ekurhuleni (Tyson et al., 1972, Goldreich, 1992). An increasing trend in heat stress and the number of heatwaves has been identified in the last decades (Ncongwane et al., 2021, van der Walt and Fitchett, 2021) and projections show further increases towards the future (Mbokodo et al., 2020). Both cities have pledged climate action plans (Ekurhuleni Metropolitan Municipality, 2015, van Weele et al., 2020, City of Johannesburg, 2021) and are members of the C40 cities initiative (Johannesburg is a steering committee member), collaborating with other cities to deliver urgent action to confront the climate crisis. Despite these actions, urban planning and design decisions are currently still based on limited low-resolution data sources, which do not adequately represent the large spatial differences in between city quarters, e.g. the contrast between high-class residential areas with a lot of urban green and impoverished densely populated counterparts (Hardy and Nel, 2015).

In this study, firstly, present and future heat stress is assessed at 30 m resolution for a large part of the Gauteng province in South Africa, comprising both the metropolitan areas of Johannesburg and Ekurhuleni. Several heat stress indicators are calculated, allowing to distinguish heat-prone regions within both cities. Secondly, meter-scale heat stress is determined by calculating the Wet Bulb Globe Temperature (WBGT), a widely applied index for heat stress measurement (Willett and Sherwood, 2012, Li et al., 2020), for individual neighborhoods. The high spatial detail enables to take into account detailed land use, radiation and individual building and tree shadows. These numerical modeling approaches allow a very detailed assessment of heat stress in the region and help in understanding micro- and local climate conditions with a goal to offer evidence-based support in climate-resilient urban planning and design. One of the main goals is to assess the effect of vegetation and shade on heat stress. In order to validate the modeling results, an extensive field monitoring campaign is performed with the help of local volunteers, which also contributed to generate climate change awareness within the community.

## 2 Materials and methods

**Fig. 1:**
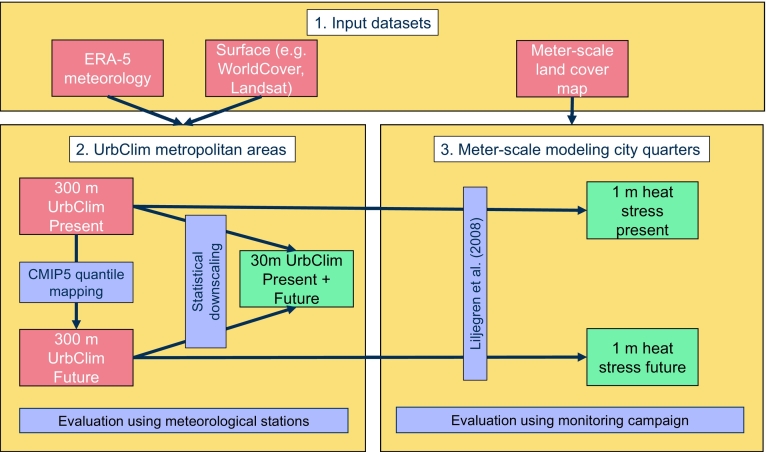
Methodological workflow. Red boxes show input datasets or temporary products. A table with details of the different input datasets is provided (Table 1). Blue boxes indicate different methodological steps, while green boxes denote the final products.

A schematic overview of the methodology used in this paper is provided and complements the text in this section (Fig. 1).

### 2.1 UrbClim setup

The urban boundary layer UrbClim numerical model is developed to ensure fast and accurate high-resolution long-term meteorological output at a city level, such as temperature, humidity, but also heat fluxes and soil parameters. The model consists of a detailed land surface scheme with simplified urban physics and a 3-D atmospheric boundary layer model. The former is based on the soil–vegetation–atmosphere scheme of De Ridder and Schayes (1997), extended for urban physics by inclusion of the inverse Stanton number. A detailed description of the UrbClim numerical model and its speed compared to full mesoscale models can be found in De Ridder et al., 2015, García-Díez et al., 2016.

The synoptic forcing at the top and lateral boundaries of the UrbClim domain is achieved from ERA-5 re-analysis of the European center for medium-range weather forecasting (ECMWF; Hersbach et al., 2020), which is available at 31 km resolution and is updated hourly. Next to state-of-the-art meteorological data, a detailed representation of land surface properties is required as input for the UrbClim numerical model. In the last years, several satellite-based products became available over Africa and they are used for the first time in an UrbClim simulation in this study. Land cover at high resolution is derived from WorldCover, a global land cover map at 10 m resolution (Zanaga et al., 2021). Combining this dataset with information from the Global Human Settlement Layer, which consists of a repository of built-up areas derived from Sentinel-2 (Corbane et al., 2021), allows to yield building fraction information for each grid cell. Soil sealing fraction is obtained from Zhang et al. (2020) who derived a global map at 30 m resolution by combining information from Landsat 8, Sentinel-1 and VIIRS in a random forest classification model. Soil texture variability is covered at 250 m spatial resolution by the dataset of Hengl and MacMillan (2019), who used statistical predictions and machine learning techniques, while Normalized Difference Vegetation Index (NDVI) maps are acquired from the Landsat 8 archive at 30 m spatial resolution at a monthly frequency using Google Earth Engine (Gorelick et al., 2017). NDVI is calculated as the ratio between the red (R) and near infrared (NIR) values (Eq. (1)):

(1) $$\mathrm{NDVI}=\frac{\mathrm{NIR}-R}{\mathrm{NIR}+R}$$

These are processed to vegetation cover fractions using the linear relationship of Gutman and Ignatov (1998, Eq. (2)):

(2) $$\mathrm{Fraction}=\frac{\mathrm{NDVI}-{\mathrm{NDVI}}_{\min}}{{\mathrm{NDVI}}_{\max}-{\mathrm{NDVI}}_{\min}}$$

${\mathrm{NDVI}}_{\max}$ and ${\mathrm{NDVI}}_{\min}$ are respectively the maximum and minimum NDVI over the domain and year. Anthropogenic heat fluxes are obtained via 30 arc-second resolution maps that are derived by Jin et al. (2019), which are based on energy demand and population numbers and thus also include for example heat emissions from air-conditioning. Lastly, terrain elevation data was gathered from the Copernicus GLO-30 Digital Elevation model dataset, which is freely available at a global scale (30 m) from the European Space Agency within the COPERNICUS program (ESA, 2020). An overview table of all input data described above is made available (Table 1).

**Table 1:** Overview of the input datasets used in UrbClim and in the meter-scale modeling.

Input dataset	Source
ERA-5 Synoptic forcing	Hersbach et al. (2020)
WorldCover Land use	Zanaga et al. (2021)
Global Human Settlement Layer built-up surfaces	Corbane et al. (2021)
Soil sealing	Zhang et al. (2020)
Soil texture	Hengl and MacMillan (2019)
Landsat 8 vegetation cover	Gutman and Ignatov (1998)
Anthropogenic heat flux	Jin et al. (2019)
Copernicus Digital Elevation Model	ESA (2020)
Building footprints and streets	Open Street Map
Google Africa Buildings	Sirko et al. (2021)
Gauteng City-Region Observatory land cover	GCRO (2022)
Digital Terrain Model Elevation-4	Airbus

**Fig. 2:**
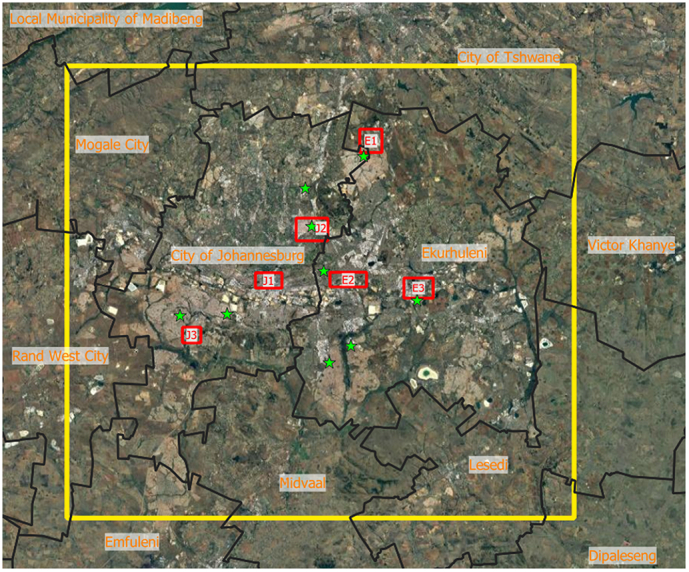
Overview of the modeling domain of UrbClim (yellow) and the six neighborhoods in which meter-scale heat stress modeling is executed. J1: Braamfontein; J2: Lombardy; J3: Soweto/Kliptown; E1: Tembisa; E2: Primrose; E3: Actonville. Locations of meteorological validation sites are denoted by green stars.

The UrbClim modeling domain covers two metropolitan city areas, Johannesburg and Ekurhuleni. This encompasses an area of 90 x 80 km (denoted in yellow in Fig. 2). Both cities are located in inland South Africa on the Highveld plateau, at an average altitude of 1750 m above sea level. In the Köppen-Geiger climate classification, it is assigned a subtropical highland climate, with a dry winter and warm summer (Cwb; Peel et al., 2007, Beck et al., 2018). In order to keep computational time within reasonable range, UrbClim simulations were executed at a spatial resolution of 300 m for a period of 20 years representing the last decades (2001–2020). A statistical downscaling technique, moving window regression (e.g. Jaberalansar et al., 2018), using the available high resolution satellite information (WorldCover land use and Landsat 8 NDVI) as predictors, is applied to obtain climate information at 30 m resolution, permitting detailed interpretations of heat stress at very local scale covering the full metropolitan areas.

The UrbClim numerical model is also used to project future climate in cities. Climate scenarios up to 2050 are obtained via statistical downscaling of the publicly available set of Global Climate Model (GCM) data within the Coupled Model Intercomparison Project 5 (CMIP5) database available on the Copernicus Climate Data Store (for an overview of the models that were used, see Table A.2). Instead of re-running the UrbClim model with CMIP5 forcing, historical time series data from the UrbClim numerical model are perturbed following the climate change signal obtained from this CMIP5 data archive using a quantile mapping bias algorithm (Olsson et al., 2009, Willems and Vrac, 2011). The result of the perturbation based statistical downscaling method consists of time series of the same length and time scale as the historical time series, but following future climate conditions. A detailed description of the procedure applied in this study can be found in Lauwaet et al. (2019, Chapter 4). Future climate simulations for the periods 2021–2040 and 2041–2060 are executed for two scenarios, Representative Concentration Pathway (RCP)4.5 (an intermediate warming scenario with emissions that peak around 2040) and RCP8.5 (worst-case climate scenario with emissions to rise throughout the 21th century). It must be noted that future urban development and growth is not included in this study. As such, the spatial profile of the city is constant in future simulations. For the anthropogenic heat flux, future projections are available until 2050 (Jin et al., 2019), which is included in the UrbClim simulations.

### 2.2 UrbClim validation

Since heat stress indicators at 30 m spatial resolution are calculated based on UrbClim results and the fact that UrbClim output (air temperature, humidity and wind speed) serves as input for the meter-scale heat stress modeling (see Section 2.3), an evaluation of its simulated urban meteorology is required.

In recent times, the South African governments have developed an air quality monitoring system with several measurement locations, mainly in city metropolitan areas, of which most provide basic meteorological information on an hourly time scale (Gwaze and Mashele, 2018). The instrumentation is calibrated yearly following ISO 17025 norms, leading to a maximum uncertainty of 0.2 °C for temperature and 2 % for relative humidity, based on the documentation of the South African National Accreditation System. This data, despite their irregular temporal variability, is quality and consistency checked since 2015 and can be accessed via https://saaqis.environment.gov.za/. Nine of these stations are located in the modeling domain and provide data within the time window 2015–2020 (Their location is visualized on Fig. 2). An evaluation of the minimum and maximum 2 m temperature and humidity simulated by UrbClim is executed. For this, we calculated the Bias, the Root Mean Square Error (RMSE) and the correlation coefficient, by comparing the station measurements with 30 m downscaled data from the pixel within the UrbClim results encompassing the station.

### 2.3 Meter-scale heat stress modeling

Several indicators to calculate heat stress are available. The Wet Bulb Globe Temperature (WBGT) is of particular interest as it uses health-related risks of heat as a basis (Heo et al., 2019), utilizes standard meteorological variables and can be calculated both in direct solar radiation and shady conditions (Lauwaet et al., 2020). International standards have been agreed to determine the physical heat stress limits for normal healthy persons depending on their type of activity or metabolic rate (ISO, 2017, Table A.3). The WBGT is calculated following the method of Liljegren et al. (2008), which is recommended for outdoor heat stress measurements by Lemke and Kjellstrom (2012) and makes use of the natural wet bulb temperature ($T_{w}$), the globe temperature ($T_{g}$) and the ambient dry bulb temperature ($T_{a}$):

(3) $$\mathrm{WBGT}=0.7T_{w}+0.2T_{g}+0.1T_{a}$$

In order to provide meter-scale calculations of heat stress, a detailed description of the land surface, buildings and trees (and their heights for shade calculations) is required. These are summarized in Table 1 and detailed below. Individual building footprints are obtained from the Google Africa Buildings dataset (Sirko et al., 2021) and Open Street Map, the latter from which also the road network is obtained. Remaining data gaps such as open spaces and vegetation were filled using the Gauteng City-Region Observatory detailed 2.5 m land cover map that is available in their GIS viewer (GCRO, 2022) which is combined and reclassified using information from the 10 m WorldCover product (Zanaga et al., 2021). Building and tree heights were obtained via Airbus Elevation-4 data, allowing us to create a very detailed land use map. By taking into account the solar zenith and azimuth angle, for each hour of each day in the year, the pattern of shadows from buildings and trees can be simulated and the sky view factor is calculated (Dirksen et al., 2019). Both direct and diffuse solar radiation, including radiation transfer though tree canopy is taken into account. Meteorological input such as temperature, humidity and wind speed are taken from the UrbClim results at 300 m. The land surface, radiative and meteorological information described above is included in the algorithm of Liljegren et al. (2008). An iterative approach is used to close the radiative balance and obtain the $T_{w}$ and $T_{g}$ components (see Eqs.  (6) and (17) in Liljegren et al., 2008) for each meter-scale pixel and hourly time step, which is ultimately utilized to calculate the meter-scale WBGT output (Eq. (3)).

The meter-scale modeling has been performed for six city quarters (Fig. 2). For each of these neighborhoods, one full day with (near) clear-sky conditions was simulated in the summer period January-February 2022. During these days, an extensive monitoring campaign was completed (see Section 2.4), which was used to evaluate the modeling results. Furthermore, for one historical heatwave, simulations are executed to better understand the spatial pattern of heat stress at the meter-scale level and distinguish differences between areas in both present and future times.

### 2.4 Monitoring campaign

A series of monitoring campaigns involving community participants were conducted, focusing on the measurement of the WBGT. While the goal of these monitoring campaigns was largely to provide reference validation data for the modeling, they were designed to also serve in their own right, by providing quantitative evidence of the cooling impact of trees (see below), and – through the involvement of community field workers – by generating community awareness, engagement and empowerment. A detailed description of the latter can be found in Appendix A.

**Fig. 3:**
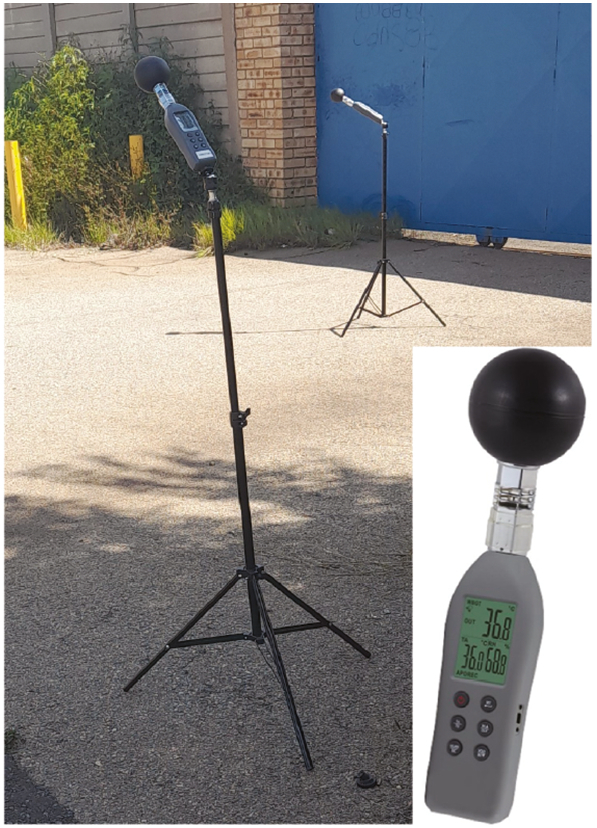
Data logger and typical setup during a campaign with one device in the sun and one in the shade.

Each of the six monitoring campaign days involved 16 portable WBGT data loggers of the type AT-HTS01 (see Fig. 3), the specification of which are available from https://www.atal.nl/media/downloads/mn/az/AT-HTS01.pdf. These loggers measure the constituents of the WBGT, such as $T_{g},T_{a}$ and the relative humidity, which can be converted to $T_{w}$ using the approach of Liljegren et al. (2008). From this, WBGT can be achieved using Eq. (3). An important constraint of the WBGT loggers is their response time of about 15 min, meaning that measurements have to be conducted in a rather static way, i.e., the WBGT logger remaining at a fixed position during approximately 30 min while recording WBGT data values.

For each of the six monitoring campaigns, we recruited 16 community participants, each of them being in charge of one of the 16 WBGT data loggers. A challenging aspect was that the community participants had to be mobilized at fairly short notice, of the order of a few days, since the monitoring campaign days were selected a few days in advance only, based on the weather forecast, considering suitable weather conditions to be characterized by cloud-free and warm conditions. The reason for focusing on cloud-free situations is related to the fact that one of our main objectives was to measure the cooling impact of trees (through shading), which obviously requires sunny conditions. Such conditions were not always met, as scattered cloud often showed up in the early afternoon, precisely at the time scheduled to conduct the measurements (since we were aiming to monitor during the hottest hours of the day). In fact, the 2021–2022 (austral) summer in South Africa was characterized by below-average air temperatures and above-average cloudiness and precipitation, which has been ascribed to a weak La Niña event taking place during this period (WMO, 2022).

The WBGT measurements were conducted in pairs, one of the devices measuring in the shade of a tree and the other receiving full solar radiation exposure at a nearby position (meters to tens of meters away), the main aim of this approach being to measure the (local) cooling impact of shade (Fig. 3). Each pair of data loggers was set up at a designated location, where a measurement covering a 30-min time lapse was conducted. Recall that such a long time lapse is required for the measurement to reach equilibrium (see above).

In each of the campaign days, every WBGT data logger was used to conduct two measurements, one extending between 1:30 and 2:00 PM, and a subsequent measurement extending between 2:30 and 3:00 PM, leaving half an hour to the community participants to move to a second designated location and set up their measurement gear there. As a result of the six campaign days, each involving 16 WBGT data loggers and two measurements taken per campaign per logger, we collected 192 data points (the locations of these measurements are denoted in Fig. A.4). These data points were compared to model results of WBGT, but also the individual components as listed in Eq. (3). The Bias, the Root Mean Square Error (RMSE) and the correlation coefficient were again calculated.

## 3 Results and discussion

### 3.1 Model evaluation

**Fig. 4:**
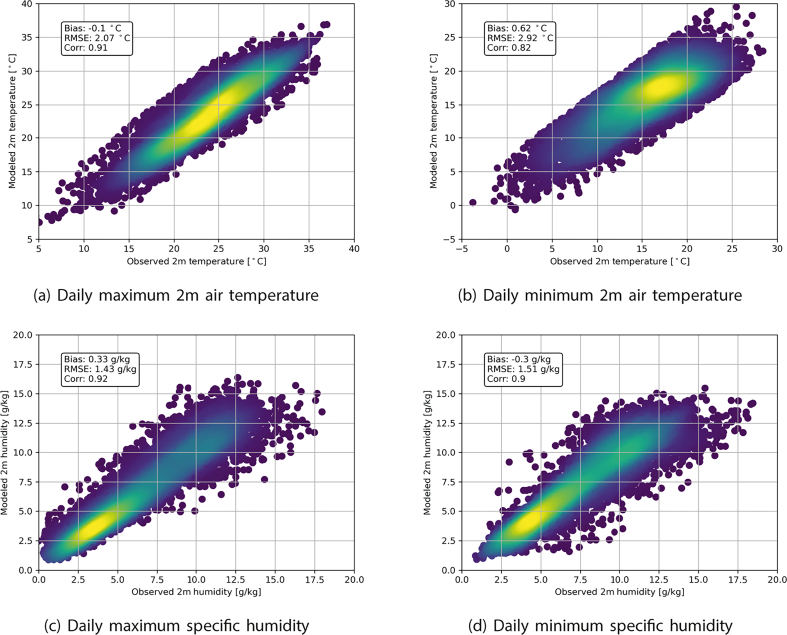
UrbClim evaluation.

Generally, a limited bias of −0.10 °C and 0.62 °C between measured and modeled maximum and minimum 2 m temperatures respectively is found (Fig. 4). A small overestimation in nighttime 2 m temperatures by UrbClim can be attributed to the fact that most meteorological observation stations are shielded by a fence with some nearby vegetation in an urban context. This vegetation, which tend to cool faster during the night, is not always represented in the land cover surface of UrbClim (which has a 30 m resolution). Higher vegetation abundance therefore accounts for cooler air temperatures during the night, attributing for the slightly higher temperatures modeled at night for some stations by UrbClim (Fig. 4b). This also explains the higher variability and RMSE for nighttime 2 m temperatures.

Humidity is generally very well simulated in the model compared to the observational network. A low bias and RMSE of respectively 0.3 g/kg and 1.5 g/kg is found for both daytime and nighttime values (Fig. 4). The high correlation coefficient of 0.9 or more indicates the accurate representation of local humidity by the model.

**Fig. 5:**
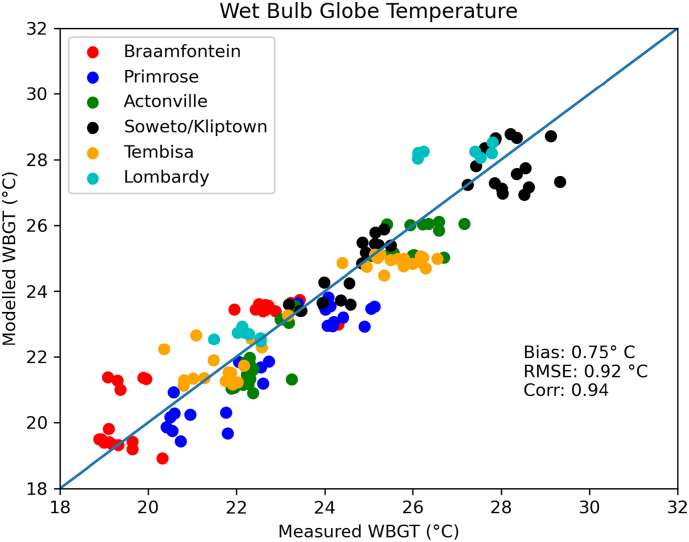
Comparison between modeled and measured Wet Bulb Globe Temperatures for the six quarters defined in Fig. 2.

For six neighborhoods, a meter-scale heat stress modeling effort and extensive monitoring campaign was executed (Fig. 2 and Section 2.4) by calculating/measuring the WBGT (see Eq. (3)). A wide range of WBGT values is captured, ranging from limited to high heat stress. A close relation between measurements and modeled values is found for all city quarters, with limited variability, high correlation (0.94) and a small structural bias (0.75 °C; Fig. 5). For each location, two clusters of measurements can be detected: one cluster with lower WBGT identified as the measurements that were obtained in the shade, and one cluster with higher WBGT showing the measurements impacted by direct solar radiation (see also Fig. 3 for the measurements setup). This distinction is well captured in the model attaining similar offsets compared to the measurements. Additionally, some variability between measurements in tree and building shadows is visible in both measurements and the model. This can be observed for example for Soweto, where a cluster around 24 °C is identified as tree shadow measurements, while the cluster at 25.5 °C is associated to measurements in the shade of buildings (Fig. 5). Generally, WBGT values in building shadows are slightly higher compared to tree shadows in both the model and measurements, showing the cooling effect of vegetation in an urban context.

**Fig. A.2:**
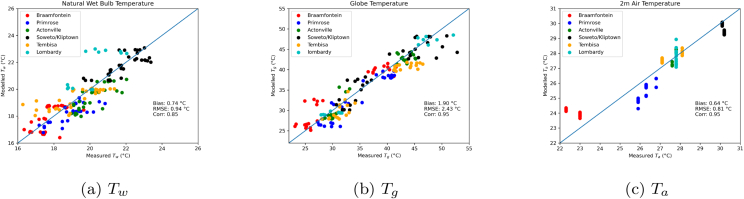
Comparison of the modeled and measured individual components of the Wet Bulb Globe Temperature for the six quarters defined in Fig. 2.

A comparison of the individual components of the WBGT is available in the Appendix (Fig. A.2). Values for $T_{w}$ are rather small (up to 24 °C) indicating that heat stress experienced in Johannesburg and Ekurhuleni is highly influenced by solar radiation and air temperatures and less by humidity. This is reflected by the high $T_{g}$ (up to 50 °C) that are modeled and measured in direct sunlight.

### 3.2 Spatial analysis of city-wide (future) thermal comfort

**Fig. 6:**
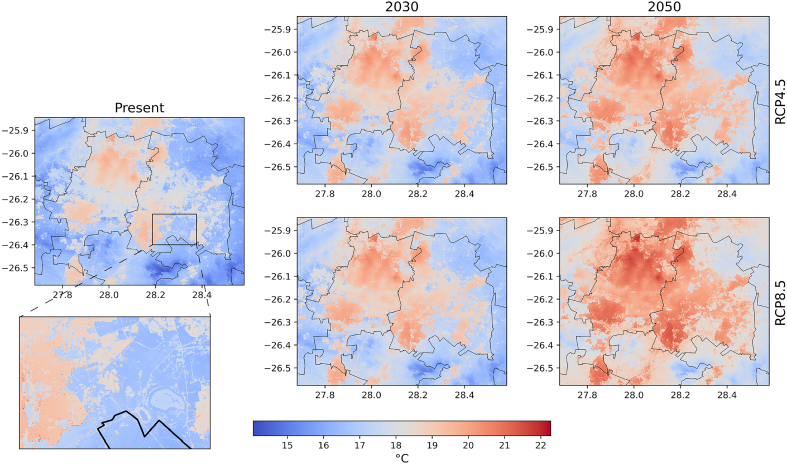
Average 2 m temperature for present (2001–2020) and future scenarios for the metropolitan areas of Johannesburg and Ekurhuleni. Inset showing the high spatial detail that is achieved.

**Fig. A.3:**
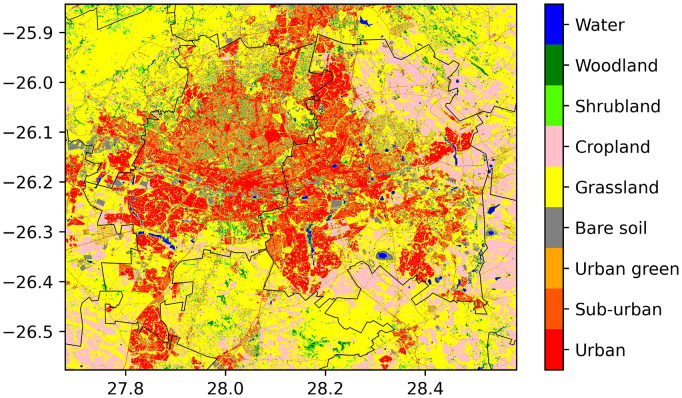
30 m land cover map for the metropolitan areas of Johannesburg and Ekurhuleni.

As both UrbClim and the meter-scale heat modeling attained reliable results compared to observations, a spatial analysis of thermal comfort experienced in Johannesburg and Ekurhuleni and at meter-scale resolution is performed. The evolution of the average 2 m temperature for two future scenarios up to 2050 at 30 m spatial resolution is shown in Fig. 6. The built-up extent of the city can easily be distinguished being several degrees (up to 5 °C) warmer than rural areas. A high resolution land cover map at 30 m spatial resolution is added to the Appendix to facilitate the comparison (Fig. A.3). The high resolution of 30 m allows to identify roads and small features, such as grouped buildings and mines, which have higher air temperatures compared to their surroundings, but also parks and vegetation patches, which exhibit cooler air temperatures (see inset in Fig. 6). These maps allow to detect at very high detail the spatial variability in heat for both metropolitan areas and permit to zoom into specific locations of interest.

Both metropolitan areas have a highly variable topography. The central part of the cities is elevated a few hundreds meters above the rest of the city, while the northern part of Johannesburg has the lowest altitudes, explaining part of the air temperature differences in Fig. 6. Towards the future, it is observed that air temperature increases by 2030 are relatively similar in an RCP4.5 and RCP8.5 scenario, with a slight increase of approximately 1 °C in 2 m temperatures over both rural and urban areas (Fig. 6). This changes towards 2050, where a clear distinction in air temperatures is observed between RCP4.5 and RCP8.5 with domain-average 2 m temperature increases of approximately 1.5 °C and 3 °C respectively compared to the present.

Apart from average 2 m temperatures, several other heat stress indicators for both present day and future scenarios have been calculated. These are listed in Table A.4 and can be accessed as both high-resolution figures and georeferenced GeoTiffs via https://doi.org/10.5281/zenodo.6394130.

**Fig. 7:**
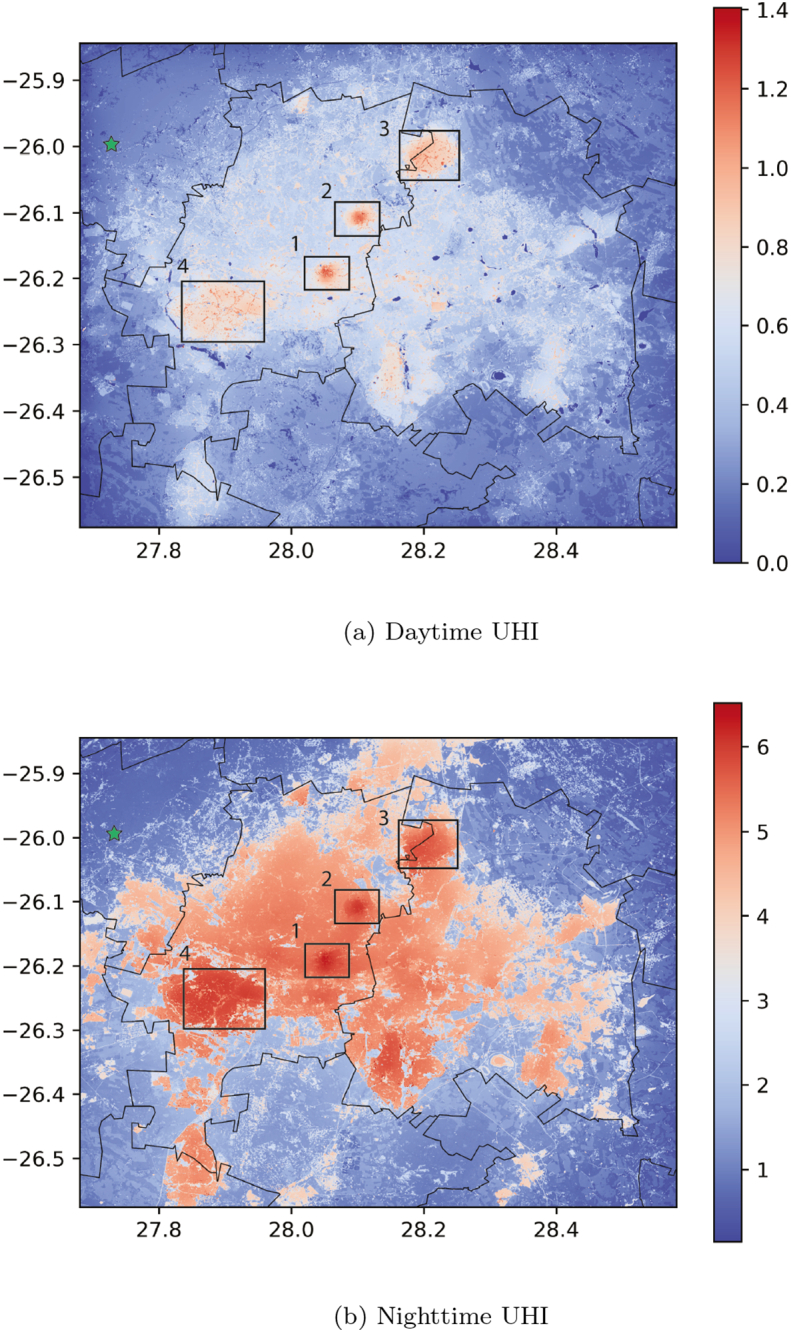
Urban heat island corrected for topography. The green star indicates the rural location used as a baseline in the calculation. 1  = Central Business District; 2  = Alexandra township; 3  = Tembisa township; 4  = Soweto township.

Due to the highly variable topography, the exact effect of urban canopy on air temperatures is more difficult to interpret. By removing topography and subtracting temperatures over the full domain with the temperature of a rural location (calculated as the first percentile temperature over grasslands in the domain; see Fig. A.3), the net effect of urban land use on air temperatures can be visualized (the so-called UHI; Fig. 7). The UHI is larger during the night compared to the day, with 2 m temperatures up to 6 °C higher during nighttime compared to rural locations. It is noted that the warmer air temperatures also persist up to several kilometers downwind the city bounds. This phenomenon, urban heat advection, is also observed in other recent modeling and observational studies (Cosgrove and Berkelhammer, 2018, Brousse et al., 2022, Dinda and Chatterjee, 2022).

In both day- and nighttime, several hot spots can be identified which are a few degrees warmer compared to other locations in the city. A first hot spot is located in the Central Business District of Johannesburg (denoted by 1 in Fig. 7). This is an area with a high building density and limited or no vegetation, accounting for the higher air temperatures simulated by the model. One would expect the urban canyon effect and the large amount of shades from the high buildings to lower air temperatures in these areas. However, this is compensated for by the large amount of sealed surfaces and higher anthropogenic heat fluxes over this area. Another neighborhood that spikes up regarding 2 m temperatures in both day- and nighttime is located in Alexandra (denoted by 2 in Fig. 7). This area was previously known for its large amount of informal settlements and backyard structures (Beall et al., 2000), but has recently undergone some transformation of spatial form in order to increase the quality of life, which was quite effective in the southern and eastern parts of this area. The oldest part of this neighborhood however, still consists mainly of densely populated informal settlements (Harrison et al., 2014, Pieterse and Owens, 2018). In this part in particular, the peak in 2 m temperatures is recorded compared to surroundings.

The townships of Tembisa and Soweto are indicated by respectively 3 and 4 on Fig. 7. Tembisa township was artificially created around 1960 in a resettlement operation. Despite the orderly structure of streets, there is a high density of buildings, with limited vegetation and lots of bare and concrete structures, typical for the poor living conditions in this area (Rakabe, 2016). In Soweto, fundamental socio-spatial transformations have taken place in the last decades. Larger formal houses are being built and infrastructure is generally well developed (Harrison and Harrison, 2014). Despite this, building density is very high and limited vegetation is present in the area. Notwithstanding the more formal housing in Soweto, a similar 2 m temperature footprint compared to Tembisa is observed (Fig. 7). This shows that the drivers of the UHI are mainly related to building density, sealed surfaces and vegetation presence. The northern parts of Johannesburg for example, have much more vegetation abundance (see Fig. A.3) and generate a lower UHI (several degrees less) compared to the rest of the city.

### 3.3 Meter-scale (future) heatwave modeling

**Fig. 8:**
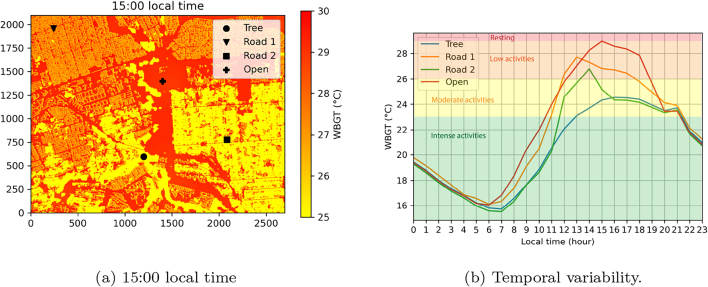
Spatial and temporal evolution of WBGT for Lombardy/Alexandra during the heatwave of 6 January 2016. The color scheme in the background shows metabolic activities for people unacclimatized to heat (Table A.3).

**Fig. A.4:**
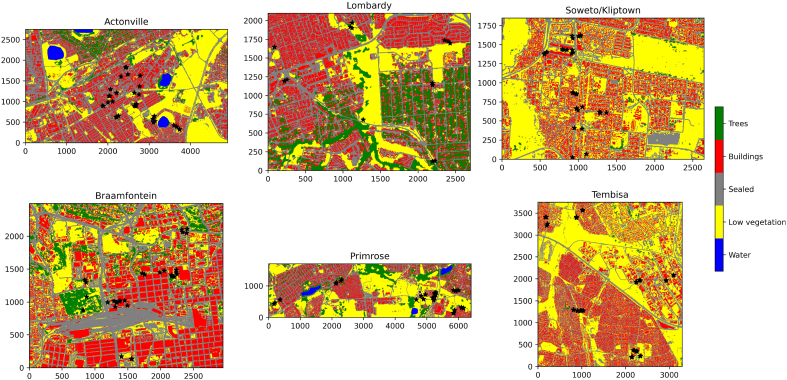
Meter-scale land cover map for the meter-scale heat modeling domains. Black stars indicate the locations of the monitoring campaign measurements.

**Fig. A.5:**
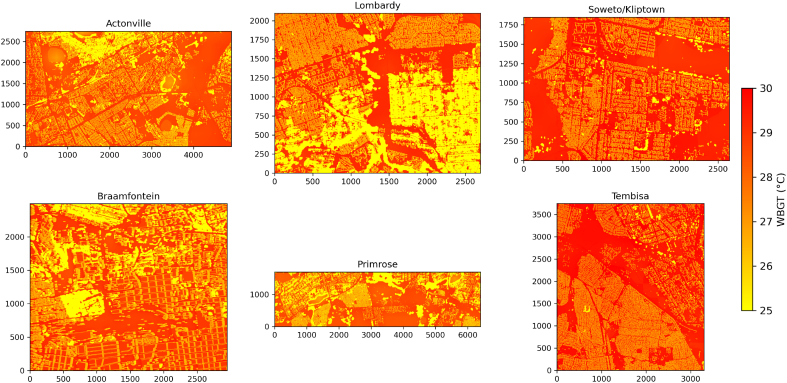
Wet Bulb Globe Temperatures for all domains specified in Fig. 2 for the heatwave of 6 January 2016 at 15.00 h local time.

Apart from metropolitan-wide results, meter-scale heat stress modeling efforts have also been performed for a selection of city quarters (Fig. 2). In order to assess the spatial and temporal variability of heat stress, the record-breaking heatwave of January 2016, reaching air temperatures up to 38 °C, is analyzed (Brimicombe et al., 2022). The peak of this heatwave, on the 6th of January 2016 is visualized for the city quarter of Lombardy/Alexandra since it consists of both a well-developed residential area and informal settlements (Fig. 8). Results for the other neighborhoods can be found in the Appendix (Fig. A.5). 3D animations can be assessed via https://bit.ly/3OeabxW. Clear spatial differences in heat stress are experienced at different locations within this neighborhood at the peak of the heatwave at 15:00 local time (Fig. 8a). In the south-east, the suburb Lombardy East is located. This area comprises of residential buildings on larger properties with a lot of vegetation and trees; a detailed land use map can be found in the Appendix (Fig. A.4). The cooling effect of trees through shade and evapotranspiration is clearly visible on the map, with WBGT values not going above 25 °C at the peak of heat stress during the day. In the north-west, the informal settlements of Alexandra are located. Dense building structures and no vegetation are present, leading to WBGT values which are several degrees warmer compared to Lombardy East. Open spaces that experience no shielding from direct sunlight record the highest WBGT values, up to 29 °C. For the other city quarters, similar results are obtained when simulating the heatwave of 6 January 2016, showing discrepancies between areas with a lot and limited vegetation (Fig. A.5). The city quarters of Tembisa and Soweto stand out most, having almost no vegetation or trees, limiting cooling by evapotranspiration and experiencing highest heat stress.

Differences in heat stress over different areas become even more clear when visualizing its temporal variability during the day (Fig. 8b). Tree shade and evapotranspiration from vegetation provide effective tools to counter heat stress (Maranga, 2021, Wong et al., 2021, Van de Walle et al., 2022). Even during the record-breaking heatwave, the WBGT stayed below 25 °C in the shade of trees during the whole day, allowing activities to be executed in their shade for people that are acclimatized to heat, while unacclimatized people could still perform basic activities (Table A.3). The WBGTs of the streets for both Lombardy East (Road 1) and Alexandra (Road 2) have also been assessed. Locations were selected that are impacted by direct sunlight up to 13:00 (for Alexandra) and 14:00 (for Lombardy East), while being located in the shade for the rest of the day. In Lombardy East, the street is surrounded by trees, while a dense building pattern of informal settlements is surrounding the street in Alexandra. This is reflected in WBGT values, which are consistently 2 °C lower in Lombardy East during the first hours of the day during direct sunlight exposure. Once both locations become shielded from direct sunlight in the early afternoon, we see a rapid decline in WBGT in Lombardy East, reaching values similar to locations that have been shielded by tree shade for the whole day within two hours. In Alexandra, the street is shielded by building shade. These buildings have warmed up in the morning and still emit heat for multiple hours. During the whole afternoon, WBGT values above 26 °C are recorded, up to 3 °C more than in Lombardy East, prohibiting moderate and intense activities for persons acclimatized to heat and limiting unacclimatized persons to resting or very basic activities (Table A.3). Only after sunset, similar heat stress values are achieved for both locations. Locations that are not shielded by any shade during the whole day experience the highest heat stress. In this case, WBGT values up to 29 °C are attained, limiting activities to the lowest effort rates during most of the afternoon.

**Fig. 9:**
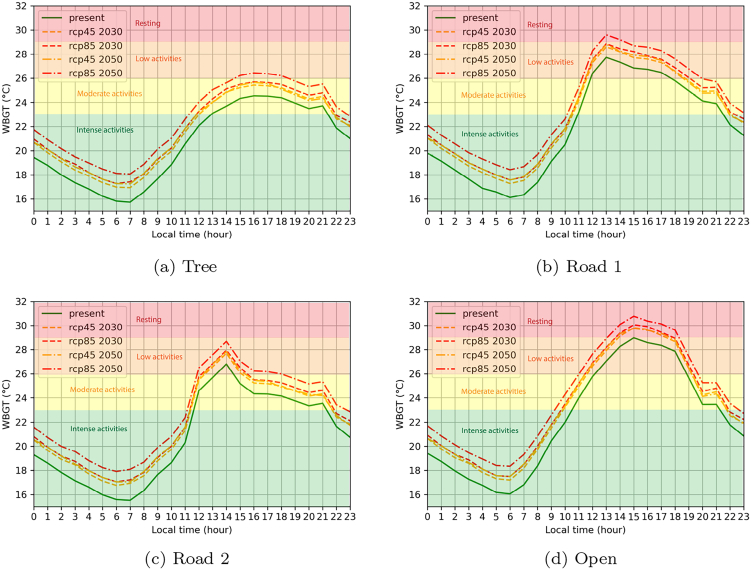
Future WBGT projection of the 6 January 2016 heatwave for the locations specified in Fig. 8. The color scheme in the background shows metabolic activities for people unacclimatized to heat (Table A.3).

Apart from present-day conditions, we also assess the future impact of the 6 January 2016 heatwave. This can be modeled by applying the quantile mapping approach as explained in Section 2.1. For the four locations selected in Fig. 8, the WBGT has been visualized for different future time frames and scenarios (Fig. 9). A first observation is that the increase in WBGT towards the future is of a lower level compared to the expected increase in near-surface air temperatures (compare with Fig. 6). This can be explained by the fact that WBGT also takes into account humidity (Eq. (3)), which is projected to decrease towards the future with concurrent rising air temperatures (Fischer and Knutti, 2013), mitigating heat stress exposure (Brouillet and Joussaume, 2019, Wouters et al., 2022). Despite the projected decrease in humidity, still a clear increase in heat stress is expected towards the future, attaining values of WBGT that are 1.5 °C higher in 2030 and up to 2.5 °C in 2050 under an RCP8.5 scenario. In practice, this will lead to more severe and longer time periods of heat stress exposure.

Increases in heat stress are uniform over all land use types. However, there are some important differences in consequences regarding human health and heat stress exposure. In tree shade for example, under present day climate conditions, moderate activities could still be executed, while in future scenarios, these might also imply health consequences. In densely-built settlements without vegetation, heat exposure becomes much more problematic (Fig. 9b). In the RCP8.5 scenario in 2050, WBGT values over 29 °C are attained from noon until 15:00 local time, limiting any kind of activity in this area without posing health risks. Open spaces should be avoided at all costs in these future climate scenarios (Fig. 9d). Where under present conditions, activities as walking could still take place, even this can lead to health problems in any of the future climates as early as 2030 for most of the afternoon.

## 4 Conclusion

Heat stress is experienced more intensely in urban areas compared to rural locations and projections indicate that climate change effects will increase the impacts on human health. Research on urban heat stress has previously been performed using different setups, ranging from meso-scale to micro-scale models, mainly in mid-latitudinal regions. These have the problem that they require respectively a large computational cost and are limited to very small domains respectively. In this study the UrbClim numerical model is applied, allowing to study the urban climate in high detail at limited computational costs for long time periods. In this study, urban heat (stress) is studied over the metropolitan areas of Johannesburg and Ekurhuleni, South Africa. Apart from the relevance of investigating urban heat stress over the African continent, both cities have implemented (and are planning) socio-spatial conversions at several neighborhoods, making them a practical and useful case study.

An extensive observational campaign was executed allowing to obtain validation data, but also to encourage the local community to engage in climate change research. The former was used to demonstrate an accurate representation of the model results. Urban areas exhibit much higher air temperatures compared to rural counterparts and based on the design of the city, hot spots of air temperatures pop up in neighborhoods of high building density and areas with limited urban green. The meter-scale heat stress modeling showed the positive effect of urban green and vegetation throughout the city. A large amount of trees is shown to be a good adaptation measure to mitigate highest heat stress exposure and has been quantified fast and accurately using our simple model approach at meter-scale resolution. The study further showed that climate change will increase heat stress over all types of land cover and action is required to avoid heat stress related health problems, certainly in locations affected by direct sunlight and with limited urban green.

The high spatial resolution of the results of our study allows to investigate urban heat stress and health-related impacts at the individual street/house level, both for the present and the future. Up to now, socio-spatial conversion projects in Johannesburg and Ekurhuleni were focused on creating improved housing conditions and attracting private investments (e.g. in Soweto). Most newly built residential areas are equipped with air-conditioning and provide better thermal insulation, still several of them lack vegetation presence, which was shown to be an important problem in several neighborhoods (Fig. A.4). Our results show that lower amounts of sealed surfaces and higher extents of urban green provide an effective measure mitigating heat stress and improving living conditions, which has to be considered in the future design of city quarters and reconversion plans of Johannesburg and Ekurhuleni.

In future work, urban planning projects can be implemented in the model, allowing to quantitatively assess the impact of changes in spatial structure on heat stress. This allows to assess the effectiveness of concrete actions with respect to opposing the negative effects of climate change and heat stress.

## CRediT authorship contribution statement

**Niels Souverijns:** Software, Validation, Formal-analysis, Investigation, Data-curation. **Koen De Ridder:** Conceptualization, Methodology, Data-curation, Project-administration, Supervision, Funding-acquisition. **Nele Veldeman:** Project-administration. **Filip Lefebre:** Conceptualization, Funding-acquisition. **Frederick Kusambiza-Kiingi:** Investigation. **Wetu Memela:** Investigation. **Nicholas K.W. Jones:** Conceptualization, Project-administration, Supervision.

## Declaration of Competing Interest

The authors declare that they have no known competing financial interests or personal relationships that could have appeared to influence the work reported in this paper.

## Figures and Tables

**Fig. A.1:**
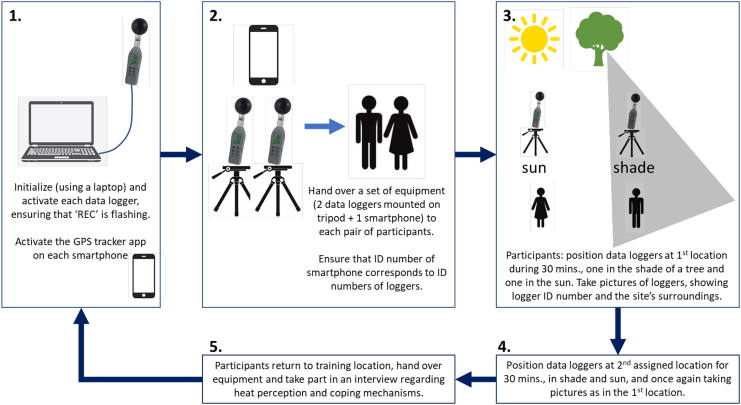
Schematic overview of the field measurements, starting with the initialization and activation of the WBGT data loggers, the hand-over to the community participants, the dual (shade-sun) measurement approach, and the return and read-out of the loggers.

**Table:** **Table A.2.** List of CMIP5 GCMs used in future UrbClim projections.

Model	Institute
BCC–CSM1-1-M	BCC, China
BNU-ESM	BNU, China
CanESM2	CCCMA, Canada
CSIRO-Mk3-6–0	CSIRO, Australia
FGOALS-g2	LASG/IAP, China
GFDL-CM3	NOAA, USA
GFDL-ESM2G	NOAA, USA
GFDL-ESM2M	NOAA, USA
INMCM4	INM, Russia
IPSL-CM5A-LR	IPSL, France
IPSL-CM5A-MR	IPSL, France
IPSL-CM5B-LR	IPSL, France
NorESM1-M	NCC, Norway

**Table:** **Table A.3.** Wet Bulb Globe Temperature reference values for acclimatized and unacclimatized people for five classes of metabolic rate (ISO, 2017). A detailed description and examples of the levels of metabolic rate can be found in ISO (2017, Annex E).

Metabolic rate	WBGT limit for persons acclimatized to heat (°C)	WBGT limit for persons unacclimitized to heat (°C)
Resting (115 W)	33	32
Low activity (180 W)	30	29
Moderate activity (300 W)	28	26
Intense activity (415 W)	26	23
Very intense activity (520 W)	25	20

**Table:** **Table A.4.** List of heat-related indicators that are calculated for the metropolitan areas of Johannesburg and Ekurhuleni for both the present and future scenarios. High-resolution pictures and georeferenced images can be accessed viahttps://doi.org/10.5281/zenodo.6394130.

1. Absolute maximum 2 m temperature during the period 2. Absolute minimum 2 m temperature during the period 3. Absolute mean 2 m temperature during the period (over all times of the day) 4. Average daily maximum 2 m temperature during the period 5. Average daily minimum 2 m temperature during the period 6. Average UHI during the day 7. Average UHI during the night 8. Number of days attaining a maximum 2 m temperature >25 °C 9. Number of days attaining a maximum 2 m temperature >30 °C 10. Number of days attaining a maximum 2 m temperature >25 °C that are caused by urban canopy (by comparing with a rural location result at same height) 11. Number of days attaining a maximum 2 m temperature >30 °C that are caused by urban canopy (by comparing with a rural location result at same height) 12. Number of nights attaining a minimum 2 m temperature >20 °C 13. Number of nights attaining a minimum 2 m temperature >25 °C 14. Number of heatwave days per year calculated following the definition of the South African Weather Service 15. Cooling degree hours (American and AirConditioning Engineers, 2021) 16. Annual number of days when the maximum 2 m temperature >90th percentile 17. Annual number of days when the maximum 2 m temperature <10th percentile 18. Annual maximum daytime coolest 2 m temperature 19. Annual number of days with at least six consecutive days when the maximum 2 m temperature >90th percentile 20. Annual minimum nighttime warmest 2 m temperature 21. Annual number of days whenthe minimum 2 m temperature >90th percentile 22. Annual number of days when the minimum 2 m temperature <10th percentile 23. Annual number of days with at least six consecutive days when the minimum 2 m temperature <10th percentile

## Data Availability

The data used in this research is made available in an open-access repository. Heat stress indicators can be downloaded as high-resolution images or georeferenced GeoTiffs via https://doi.org/10.5281/zenodo.6394130. 3D-animations of heat stress can be retrieved via https://bit.ly/3OeabxW.

## Appendix A Community participation strategy

In this section, we would like to outline the strategy used to recruit, engage and involve local community members in the research project.

For the recruitment process we worked with local community leaders and ward councilors. Together with them we agreed on the logistics, training location and the identification of suitable heat monitoring locations. The emphasis was placed on working with and recruiting women. Overall, we also had a considerable participation from youth.

Each monitoring campaign day started with a training and information session taking place at a central location (government building, church, community center, …) in the form of a workshop, where participants received general information regarding climate change and its impacts, and specific information regarding the context of the monitoring campaigns and their role in the urban heat mapping exercise. The field work was explained in terms of procedure, equipment and the planning of the day. As a final training activity, a practical hands-on demonstration was given, during which the participants learned to manipulate the equipment.

After the training session, in the early afternoon, the community participants went out into the field to conduct the measurements. A schematic overview of the field measurements is depicted in Fig. A.1. Prior to leaving for the field, they received fluorescent safety jackets, a sun protection head cover and a bottle of drinking water.

To get community participants more involved in the project, they were requested to take manual readings of the data loggers every fifteen minutes during the 30 min of measuring heat stress. The first reading was taken at the time of placing the WBGT in position, then readings were done at every 15 min interval, which made it three manual readings taken at each location. These manual readings were done in addition to the WBGT measurements. The community participants took pictures with smartphones in order to document the surroundings of the measurement locations. More importantly, the smartphones’ cameras were set to contain location information (GPS latitude and longitude), allowing to position the measurement data. This was required given that one of the purposes of acquiring the WBGT data was model validation, for which precise location information of the measurement sites is of paramount importance.

Upon their return to the training location, the community participants were taken through a reflection session, during which they were debriefed and asked about their impressions and difficulties encountered, among other.
